# Mood Disorders and Risk of Lung Cancer in the EAGLE Case-Control Study and in the U.S. Veterans Affairs Inpatient Cohort

**DOI:** 10.1371/journal.pone.0042945

**Published:** 2012-08-07

**Authors:** David E. Capo-Ramos, Ying Gao, Jay H. Lubin, David P. Check, Lynn R. Goldin, Angela C. Pesatori, Dario Consonni, Pier Alberto Bertazzi, Andrew J. Saxon, Andrew W. Bergen, Neil E. Caporaso, Maria Teresa Landi

**Affiliations:** 1 Division of Cancer Epidemiology and Genetics, National Cancer Institute, National Institutes of Health, Bethesda, Maryland, United States of America; 2 EPOCA, Epidemiology Research Center, Universita' degli Studi di Milano, Milan, Italy; 3 Unit of Epidemiology, Fondazione IRCCS Ca' Granda - Ospedale Maggiore Policlinico, Milan, Italy; 4 Veterans Affairs Puget Sound Health Care System and Addiction Psychiatry Residency Program, Seattle, Washington, United States of America; 5 University of Washington, Seattle, Washington, United States of America; 6 Molecular Genetics Program, Center for Health Sciences, SRI International, Menlo Park, California, United States of America; The University of Texas M. D. Anderson Cancer Center, United States of America

## Abstract

**Background:**

Mood disorders may affect lung cancer risk. We evaluated this hypothesis in two large studies.

**Methodology/Principal Findings:**

We examined 1,939 lung cancer cases and 2,102 controls from the Environment And Genetics in Lung cancer Etiology (EAGLE) case-control study conducted in Italy (2002–2005), and 82,945 inpatients with a lung cancer diagnosis and 3,586,299 person-years without a lung cancer diagnosis in the U.S. Veterans Affairs Inpatient Cohort (VA study), composed of veterans with a VA hospital admission (1969–1996). In EAGLE, we calculated odds ratios (ORs) and 95% confidence intervals (CI), with extensive adjustment for tobacco smoking and multiple lifestyle factors. In the VA study, we estimated lung cancer relative risks (RRs) and 95% CIs with time-dependent Poisson regression, adjusting for attained age, calendar year, hospital visits, time within the study, and related previous medical diagnoses. In EAGLE, we found decreased lung cancer risk in subjects with a personal history of mood disorders (OR: 0.59, 95% CI: 0.44–0.79, based on 121 lung cancer incident cases and 192 controls) and family history of mood disorders (OR: 0.62, 95% CI: 0.50–0.77, based on 223 lung cancer cases and 345 controls). The VA study analyses yielded similar results (RR: 0.74, 95% CI: 0.71–0.77, based on 2,304 incident lung cancer cases and 177,267 non-cancer person-years) in men with discharge diagnoses for mood disorders. History of mood disorders was associated with nicotine dependence, alcohol and substance use and psychometric scales of depressive and anxiety symptoms in controls for these studies.

**Conclusions/Significance:**

The consistent finding of a relationship between mood disorders and lung cancer risk across two large studies calls for further research into the complex interplay of risk factors associated with these two widespread and debilitating diseases. Although we adjusted for smoking effects in EAGLE, residual confounding of the results by smoking cannot be ruled out.

## Introduction

Tobacco smoking, and other environmental and genetic factors have all been implicated in lung cancer etiology [1]. Psychiatric conditions have been hypothesized to have a relationship to lung cancer risk, but the association is controversial [2], [3].

Mood disorders, mainly unipolar and bipolar depression, are the most common severe adult mental disorders and the most important psychiatric causes of disability and morbidity worldwide [4]. According to 2004 World Health Organization data, at any one time, 151.2 and 29.5 million people may be suffering from unipolar and bipolar depression, respectively [5].

Mood disorders, particularly depression, have been proposed as risk factors for cancer through diverse mechanisms, including effects on the immune system mediated through chronic stress, and associations with other risk factors such as smoking, poor diet, and increased exposure to infectious agents [2]. Common etiologies, genetic or pharmacological, have been proposed for the consistent positive bidirectional associations between depression and smoking [6], [7]. A common genetic predisposition to both mood disorders and cancer has also been proposed [8].

Previous studies have investigated the relationship between mood disorders and lung cancer incidence, with mixed results, complicated by limited ability to control for potential confounders, such as tobacco smoking and sample size. The majority have found no significant associations. The diversity of study designs, including assessment, diagnostic criteria, and detailed information on risk factors makes comparison across studies challenging, and sample sizes and corresponding person-years of follow up may have limited power in some settings [9], [10], [11], [12], [13], [14], [15], [16], [17].

In order to examine the relations between mood disorders and lung cancer, we investigated the association between them in two large studies: the Environment And Genetics in Lung cancer Etiology (EAGLE) study from the Lombardy region of Italy [18] and the U.S. Veterans Affairs Inpatient Cohort Study (VA study), including over 3.6 million adult White veteran men [19]. Unexpectedly, we found that lung cancer risk was inversely associated with both family history of mood disorders in any first degree-relative and personal history of mood disorders in the EAGLE study, and with a discharge diagnosis for mood disorders in the VA study.

## Results

### EAGLE Study

The analyses included 1,939 lung cancer cases and 2,102 controls. Sex, age and residence were not substantially different between cases and controls since they were frequency matching variables. Compared to controls, lung cancer cases tended to be less educated, less likely to be married or cohabitating, and more likely to be heavy drinkers and have higher smoking rates, e.g., higher intensity (packs per day) and longer duration (years) (Table S1). Personal history of mood disorders requiring medication or hospitalization was diagnosed in 121 (6.2%) lung cancer cases and 192 (9.1%) controls (92% provided information on their age or year of mood disorder diagnosis) (Table 1). Women were almost twice as likely to report mood disorders as men. Subjects with a family history of mood disorders, cases with no education, current smoker cases and never smoker controls were more likely to have a personal history of mood disorders. Former smoker cases and controls had a lower proportion of mood disorders. A personal history of mood disorders was associated with increased smoking duration (years) and fewer years since quitting smoking in both cases and controls (Table 1). Overall, 223 (11.5%) cases, and 345 (16.4%) controls had a first-degree relative with a previous diagnosis of mood disorders (Table 2). As expected, in control subjects, personal or family history of mood disorders was associated with depressive symptoms assessed by the Center for Epidemiological Studies Depression Scale (CES-D) [20] and Hospital Anxiety and Depression Scale (HADS) [21], and with nicotine dependence as assessed by the Fagerström Test for Nicotine Dependence (FTND) [22] (Table 3).

**Table 1:** Numbers and percentages of cases and controls with a personal history of mood disorders by demographic and behavioral characteristics in the EAGLE Study, Italy, 2002–2005.

Characteristics	Personal history of mood disorders			
	Lung cancer cases		Controls	
	Yes (n = 121)	No (n = 1,818)	Yes (n = 192)	No (n = 1,910)
	n (%)	n (%)	n (%)	n (%)
**Sex**				
Males	77 (63.6)	1,455 (80.0)	113 (58.9)	1,493 (78.2)
Females	44 (36.4)	363 (20.0)	79 (41.2)	417 (21.8)
**Age (years)**				
30–39	1 (0.8)	11 (0.6)	0 (0.0)	17 (0.9)
40–49	4 (3.3)	62 (3.4)	5 (2.6)	94 (4.9)
50–59	28 (23.1)	316 (17.4)	40 (20.8)	384 (20.1)
60–69	49 (40.5)	716 (39.4)	72 (37.5)	779 (40.8)
70–80	39 (32.2)	713 (39.2)	75 (39.1)	636 (33.3)
**Residence**				
Brescia	17 (14.1)	230 (12.7)	18 (9.4)	229 (12.0)
Milano	86 (71.1)	1,189 (65.4)	135 (70.3)	1,290 (67.5)
Monza	7 (5.8)	125 (6.9)	8 (4.2)	109 (5.7)
Pavia	5 (4.1)	123 (6.8)	16 (8.3)	112 (5.9)
Varese	6 (5.0)	151 (8.3)	15 (7.8)	170 (8.9)
**Any family history of mood disorders**				
Yes	34 (28.1)	189 (10.4)	66 (34.4)	279 (14.6)
No/Unknown	87 (71.9)	1,629 (89.6)	126 (65.6)	1,631 (85.4)
**Cigarette status (lifetime)**				
Never	17 (14.1)	115 (6.3)	75 (39.1)	604 (31.6)
Former	38 (31.4)	800 (44.0)	69 (35.9)	833 (43.6)
Current	66 (54.6)	903 (49.7)	48 (25.0)	473 (24.8)
**Cigarette intensity (packs/day)** a	1.00 (0.75–1.35)	1.00 (0.75–1.50)	0.75 (0.48–1.00)	0.75 (0.48–1.00)
**Cigarette duration (years)** a	46.5 (36.5–52.5)	44.0 (36.0–51.0)	36.0 (23.0–45.0)	32.5 (21.0–44.0)
**Years since quitting cigarettes** a	7.5 (2.0–18.0)	10.0 (3.0–19.0)	18.0 (6.0–30.0)	20.0 (12.0–29.0)
**Alcohol (grams)**				
0–4.9 g/day	31 (28.2)	327 (19.9)	52 (27.5)	423 (22.8)
5–14.9 g/day	13 (11.8)	239 (14.6)	38 (20.1)	367 (19.7)
15–29.9 g/day	27 (24.6)	409 (24.9)	44 (23.3)	462 (24.9)
30–59.9 g/day	32 (29.1)	491 (29.9)	40 (21.2)	532 (28.6)
> = 60 g/day	7 (6.4)	174 (10.6)	15 (7.9)	75 (4.0)
**Education level**				
Non-educated^b^	12 (10.0)	100 (5.5)	9 (4.7)	80 (4.2)
Elementary school	30 (25.0)	722 (39.7)	52 (27.1)	520 (27.2)
Middle/High School	71 (59.2)	903 (49.7)	115 (59.9)	1,066 (55.8)
University Degree	7 (5.8)	93 (5.1)	16 (8.3)	244 (12.8)
**Marital status**				
Married or Cohabitating	86 (71.1)	1,407 (77.4)	142 (74.0)	1,595 (83.5)
Single/Separated/Widow/Divorced	35 (28.9)	411 (22.6)	50 (26.0)	315 (16.5)

**Abbreviation:** EAGLE, Environment And Genetics in Lung cancer Etiology.

a Median (inter-quartile range).

b “Non-educated” subjects are those who did not complete the elementary school.

**Note:** Numbers of participants may not sum to total due to missing data.

**Table 2:** Numbers and percentages of cases and controls with a first-degree relative (mother, father, siblings, or children) with history of mood disorders by demographic and behavioral characteristics in the EAGLE Study, Italy, 2002–2005.

Characteristics	Family history of mood disorders			
	Lung cancer cases		Controls	
	Yes	No/Unknown	Yes	No/Unknown
	(n = 223)	(n = 1,716)	(n = 345)	(n = 1,757)
	n (%)	n (%)	n (%)	n (%)
**Sex**				
Males	164 (73.5)	1,368 (79.7)	246 (71.3)	1,360 (77.4)
Females	59 (26.5)	348 (20.3)	99 (28.7)	397 (22.6)
**Age (years)**				
30–39	0 (0.0)	12 (0.7)	2 (0.6)	15 (0.9)
40–49	8 (3.6)	58 (3.4)	20 (5.8)	79 (4.5)
50–59	52 (23.3)	292 (17.0)	74 (21.5)	350 (19.9)
60–69	85 (38.1)	680 (39.6)	142 (41.2)	709 (40.4)
70–80	78 (35.0)	674 (39.3)	107 (31.0)	604 (34.4)
**Residence**				
Brescia	25 (11.2)	222 (12.9)	43 (12.5)	204 (11.6)
Milano	170 (76.2)	1,105 (64.4)	229 (66.4)	1,196 (68.1)
Monza	13 (5.8)	119 (6.9)	17 (4.9)	100 (5.7)
Pavia	11 (4.9)	117 (6.8)	25 (7.3)	103 (5.9)
Varese	4 (1.8)	153 (8.9)	31 (9.0)	154 (8.8)
**Cigarette status (lifetime)**				
Never	18 (8.1)	114 (6.6)	100 (29.0)	579 (33.0)
Former	93 (41.7)	745 (43.4)	149 (43.2)	753 (42.9)
Current	112 (50.2)	857 (49.9)	96 (27.8)	425 (24.2)
**Cigarette intensity (packs/day)** a	1.00 (0.75–1.50)	1.00 (0.75–1.50)	0.75 (0.46–1.00)	0.75 (0.48–1.00)
**Cigarette duration (years)** a	44.0 (35.0–52.0)	45.0 (36.0–52.0)	32.0 (22.0–44.0)	33.0 (21.0–44.0)
**Years since quitting cigarettes** a	12.0 (3.0–20.0)	10.0 (3.0–18.0)	19.0 (8.0–28.0)	20.0 (12.0–30.0)
**Alcohol (grams)**				
0–4.9 g/day	48 (23.9)	310 (20.0)	86 (25.5)	389 (22.7)
5–14.9 g/day	33 (16.4)	219 (14.1)	70 (20.8)	335 (19.6)
15–29.9 g/day	41 (20.4)	395 (25.5)	83 (24.6)	423 (24.7)
30–59.9 g/day	61 (30.4)	462 (29.8)	84 (24.9)	488 (28.5)
> = 60 g/day	18 (9.0)	163 (10.5)	14 (4.2)	76 (4.4)
**Education level**				
Non-educated^b^	13 (5.8)	99 (5.8)	8 (2.3)	81 (4.6)
Elementary school	72 (32.3)	680 (39.7)	87 (25.2)	485 (27.6)
Middle/High School	125 (56.1)	849 (49.5)	212 (61.5)	969 (55.2)
University Degree	13 (5.8)	87 (5.1)	38 (11.0)	222 (12.6)
**Marital status**				
Married or Cohabitating	176 (78.9)	1,317 (76.8)	282 (81.7)	1,455 (82.8)
Single/Separated/Widow/Divorced	47 (21.1)	399 (23.3)	63 (18.3)	302 (17.2)

**Abbreviation:** EAGLE, Environment And Genetics in Lung cancer Etiology.

a Median (inter-quartile range).

b “Non-educated” subjects are those who did not complete the elementary school.

**Note:** Numbers of participants may not sum to total due to missing data.

**Table 3:** Odds ratios (95% confidence intervals) of personal or family history of mood disorders among controls (n = 2,046) by mood symptoms and measures of nicotine dependence, EAGLE Study, Italy, 2002–2005.

Behavioral characteristics	Personal history of mood disorders^a^			Family history of mood disorders^a^		
	Yes	No	Adjusted model	Yes	No/Unknown	Adjusted model
	(n = 189)	(n = 1857)		(n = 337)	(n = 1709)	
	n (%)	n (%)	OR (95% CI)	n (%)	n (%)	OR (95% CI)
**CES-D (symptoms during last week)** b						
<1 day	80 (42.3)	1399 (75.3)	1.00	219 (65.0)	1260 (73.7)	1.00
1–2 days	58 (30.7)	359 (19.3)	2.34 (1.60–3.44)	81 (24.0)	336 (19.7)	1.36 (1.02–1.83)
3–4 days	31 (16.4)	65 (3.5)	6.46 (3.79–11.02)	25 (7.4)	71 (4.2)	1.91 (1.17–3.11)
5–7 days	20 (10.6)	34 (1.8)	8.39 (4.23–16.65)	12 (3.6)	42 (2.5)	1.47 (0.75–2.90)
**HADS – Depression** c						
Normal	108 (57.5)	1352 (73.1)	1.00	223 (66.6)	1237 (72.6)	1.00
Borderline	51 (27.1)	365 (19.7)	1.61 (1.11–2.34)	83 (24.8)	333 (19.6)	1.37 (1.03–1.82)
Depressed	29 (15.4)	133 (7.2)	2.29 (1.41–3.73)	29 (8.7)	133 (7.8)	1.16 (0.75–1.78)
Missing Info	1 (0.5)	7 (0.4)	NA	2 (0.6)	6 (0.4)	NA
**HADS – Anxiety**						
Normal	103 (54.5)	1444 (78.1)	1.00	232 (69.1)	1315 (77.3)	1.00
Borderline	38 (20.1)	294 (15.9)	1.54 (1.01–2.33)	61 (18.2)	271 (15.9)	1.23 (0.89–1.69)
Anxious	48 (25.4)	111 (6.0)	5.35 (3.44–8.30)	43 (12.8)	116 (6.8)	1.99 (1.35–2.93)
Missing Info	0 (0.0)	8 (0.4)	NA	1 (0.3)	7 (0.4)	NA
**FTND** d						
Light [<4 pts]	65 (34.4)	823 (44.3)	1.00	154 (45.7)	734 (43.0)	1.00
Moderate [4–6 pts]	31 (16.4)	322 (17.3)	1.49 (0.87–2.57)	61 (18.1)	292 (17.1)	1.00 (0.69–1.46)
Heavy [7–10 pts]	19 (10.1)	123 (6.6)	2.03 (0.98–4.23)	24 (7.1)	118 (6.9)	0.98 (0.56–1.72)
Never Smokers	74 (39.2)	589 (31.7)	NA	98 (29.1)	565 (33.1)	NA

**Abbreviations:** OR, odds ratio; CI, confidence interval; NA, not applicable; EAGLE, Environment And Genetics in Lung cancer Etiology.

a Adjusted ORs for sex, age, residence, education level, marital status, time-weighted mean alcohol consumption (grams/day), smoking status, years smoking regularly, mean cigarettes per day, years since quitting cigarettes, and the interaction between MD and smoking status.

b Center for Epidemiologic Studies – Depression.

c Hospital Anxiety & Depression Scale.

d Fagerström Test for Nicotine Dependence.

**Note:** Numbers of participants may not sum to total due to missing data.

There was a significant inverse association of lung cancer with a personal history of mood disorders (OR_personal_ = 0.67, 95% CI: 0.53–0.85) or with a history of mood disorders in any first-degree relative (OR_family_ = 0.67, 95% CI: 0.56–0.81) (Table 4). These associations strengthened after further adjustment for smoking-related and alcohol consumption-related variables, education level and marital status. Similar results were observed in subjects with a family history of mood disorders in any first degree relative and in individuals with a positive family history but no personal history of mood disorders. Subjects with both a personal and a family history of mood disorders showed the greatest reduction (OR_both_ = 0.51, 95% CI: 0.31–0.85). The estimates were essentially unchanged after adjusting for smoking in any first degree relative or excluding subjects (0.57% cases and 0.76% controls) who reported a personal history of mood disorders but did not recall the date when they began treatment or hospitalization (data not shown). The Likelihood Ratio Test (LRT) showed a suggestive, but not significant, interaction between smoking status (current, former and never) and personal (*P*-value, LRT for interaction = 0.26) or family (*P*-value, LRT for interaction = 0.11) history of mood disorders (Table S2). No other interactions were identified between the covariates in the adjusted model, history of mood disorders and lung cancer risk.

**Table 4:** Numbers and percentages of cases and controls, and risk estimates for lung cancer by categories of personal or family history in the EAGLE Study, Italy, 2002–2005.

	History of mood disorders					
Mood disorders status	Lung cancer cases		Controls		Minimally adjusted^a^	Fully adjusted^b^
	(n = 1,939)		(n = 2,102)			
	Yes	No	Yes	No		
	n (%)	n (%)	n (%)	n (%)	OR (95% CI)	OR (95% CI)
Personal history	121 (6.2)	1,818 (93.8)	192 (9.1)	1,910 (90.9)	0.67 (0.53–0.85)	0.59 (0.44–0.79)
Any first degree relative history	223 (11.5)	1,716 (88.5)	345 (16.4)	1,757 (83.6)	0.67 (0.56–0.81)	0.62 (0.50–0.77)
Personal with no/unknown family history	87 (4.5)	1,852 (95.5)	126 (6.0)	1,976 (94.0)	0.75 (0.56–0.99)	0.65 (0.46–0.92)
Family with no personal history	189 (9.8)	1,750 (90.3)	279 (13.3)	1,823 (86.7)	0.72 (0.59–0.87)	0.67 (0.53–0.85)
Both personal & family history	34 (1.8)	1,905 (98.3)	66 (3.1)	2,036 (96.9)	0.57 (0.37–0.86)	0.51 (0.31–0.85)
Mother with history	61 (3.3)	1,787 (96.7)	112 (5.5)	1,911 (94.5)	0.61 (0.44–0.84)	0.66 (0.45–0.96)
Father with history	24 (1.3)	1,792 (98.7)	50 (2.5)	1,934 (97.5)	0.55 (0.33–0.89)	0.58 (0.32–1.06)
Any sibling's history	104 (6.3)	1,556 (93.7)	167 (9.4)	1,611 (90.6)	0.65 (0.50–0.84)	0.59 (0.43–0.81)
Any children' history	59 (3.6)	1,575 (96.4)	91 (5.1)	1,706 (94.9)	0.70 (0.50–0.98)	0.57 (0.38–0.86)

**Abbreviations:** OR, odds ratio; CI, confidence interval; EAGLE, Environment And Genetics in Lung cancer Etiology.

a Adjusted for sex, age and residence.

b Adjusted for sex, age, residence, smoking status, years smoking regularly, mean cigarettes per day, years since quitting cigarettes, time weighted mean alcohol consumption (grams/day), education level and marital status.

**Note:** Numbers of participants may not sum to total due to missing data.

The inverse associations with history of mood disorders were greater in current (OR_personal_ = 0.56; OR_family_ = 0.53) and former (OR_personal_ = 0.48; OR_family_ = 0.68) smokers than in never smokers (OR_personal_ = 0.97; OR_family_ = 0.89), although homogeneity of ORs was not formally rejected (Table S2). Similarly, the inverse association was most pronounced in individuals who smoked >20 pack-years (Table S3). Sex did not modify the associations between history of mood disorders and lung cancer (OR_personal_ = 0.61; OR_family_ = 0.61, for males; OR_personal_ = 0.58; OR_family_ = 0.66 for females; Table S4). Personal or family history of mood disorders did not significantly differ by lung cancer histological type or tumor grade (Table S5).

### VA Study

Between 1969 and 1996, we identified 82,945 (2.3%) and 3,586,299 (97.7%) out of 3,669,244 white veterans with an inpatient hospitalization for lung cancer and for conditions other than lung cancer, respectively, at VA hospitals. The mean year of entry was 1980 and the mean age of entry was 51.3 years.

Overall, 2,304 lung cancer cases and 177,267 non-cancer patient person-years had a previous discharge diagnosis of any mood disorders. Veterans hospitalized with mood disorders had a significantly lower risk (RR: 0.74, 95% CI: 0.71–0.78) of lung cancer, after adjustment for number of visits, age, calendar time and latency, smoking related conditions (i.e., COPD, alcohol and drug dependence and abuse and schizophrenia). The associations were slightly stronger in subjects without smoking-related conditions (Table 5). As expected, in veterans without lung cancer, the frequency of alcohol dependence and abuse, substance dependence and abuse, and schizophrenia was higher in subjects with mood disorders (Table 5). No major differences were observed when we stratified the analyses by year of hospitalization discharge, although results were slightly stronger in the ICD-9 group, where adjustments benefitted from more stringent clinical criteria (Table 6). Further adjustment for stroke and ischemic heart disease did not modify the results (RR: 0.74, 95% CI: 0.71–0.77). In addition, we examined other cancer types and did not observe a consistent pattern of association, although mood disorders-related protection was more frequent in smoking-related cancers (Table S6).

**Table 5:** Relative risks and 95% confidence intervals for lung cancer overall and by other medical conditions in the United States Veterans Affairs Inpatient Cohort: White males (n = 3,669,224) with at least one hospital admission between July 1, 1969 and September 30, 1996.

	History of Mood Disorders^a^				
	Lung cancer patients		Non-cancer patients		
Medical conditions	(number)		(person-years)		Adjusted model^b^
	Yes	No	Yes	No	
	n (%)^c^	n (%)^c^			RR (95% CI)
Overall	2,304 (100)	80,641 (100)	177,267	3,409,032	0.74 (0.71–0.78)
COPD^d^					
Yes	1,070 (46.4)	28,148 (34.9)	37,577	576,941	0.82 (0.77–0.88)
No	1,234 (53.6)	52,493 (65.1)	139,690	2,832,091	0.68 (0.64–0.71)
Alcohol dependence and abuse^e^					
Yes	1,176 (51.0)	23,413 (29.0)	88,048	812,786	0.79 (0.75–0.84)
No	1,128 (49.0)	57,228 (71.0)	89,219	2,596,246	0.67 (0.63–0.71)
Substance dependence and abuse^f^					
Yes	206 (8.9)	1,000 (1.2)	44,537	170,754	0.84 (0.72–0.97)
No	2,098 (91.1)	79,641 (98.8)	132,730	3,238,278	0.72 (0.68–0.75)
Schizophrenia^g^					
Yes	674 (29.3)	3,881 (4.8)	47,914	168,820	0.77 (0.71–0.84)
No	1,630 (70.8)	76,760 (95.2)	129,353	3,240,212	0.71 (0.67–0.74)

**Abbreviations:** RR, relative risk; CI, confidence interval; COPD, Chronic Obstructive Pulmonary Disease; ICD, International Classification of Disease.

a ICD-8 & ICD-9, code 296 which includes depression and bipolar I disease.

b Adjusted for number of visits, age, latency, calendar time, and by the stratifying variables (COPD, alcohol and substance dependence and abuse, and schizophrenia) when appropriate.

c Percentage of participants with mood disorders within each medical condition.

d ICD-8 & ICD-9, codes 490–492.

e ICD-8 & ICD-9, codes 291, 303, 305.0, 535.3, 571.0–571.3, 980.0.

f ICD-8 & ICD-9, codes 304–305.

g ICD-8 & ICD-9, code 295.

**Note:** Numbers of participants may not sum to total due to missing data.

**Table 6:** Relative risks and 95% confidence intervals for lung cancer overall and by period of discharge from the United States Veterans Affairs Inpatient Cohort: White males with at least one hospital admission between July 1, 1969, and September 30, 1996.

Model	Lung cancer cases with mood disorders^a^		
	ICD-8	ICD-9	All
	[1969–1979]	[1980–1996]	
	(n = 1,617)	(n = 687)	(N = 2,304)
	RR (95% CI)	RR (95% CI)	RR (95% CI)
Model adjusted for number of visits, attained age, calendar time and latency (Basic Model)	0.76 (0.73–0.81)	0.69 (0.63–0.74)	0.74 (0.71–0.77)
Basic model further adjusted for alcohol^b^ and drug^c^ dependence and abuse	0.75 (0.71–0.79)	0.67 (0.62–0.72)	0.72 (0.69–0.75)
Basic model further adjusted for alcohol^b^ and drug^c^ dependence and abuse, COPD^d^ and schizophrenia^e^	0.77 (0.73–0.81)	0.70 (0.64–0.76)	0.74 (0.71–0.78)

**Abbreviations:** RR, relative risk; CI, confidence interval; COPD, Chronic Obstructive Pulmonary Disease; ICD, International Classification of Disease.

a ICD-8 & ICD-9, code 296; which includes depression and bipolar disease.

b ICD-8 & ICD-9, codes 291, 303, 305.0, 535.3, 571.0–571.3, 980.0.

c ICD-8 & ICD-9, codes 304–305.

d ICD-8 & ICD-9, codes 490–492.

e ICD-8 & ICD-9, code 295.

**Note:** Numbers of participants may not sum to total due to missing data.

As expected, lung cancer risk increased with age at study entry, with numbers of hospital visits (which could be partially due to subclinical lung cancer), COPD or alcohol abuse (Table S7). In contrast, lung cancer risk decreased with the number of years of follow-up and among those who had a date of first hospitalization in the VA in the last period of follow-up (Table S7). We conducted the same analyses also excluding subjects within the last categories of Years of follow-up (15+ years) or Date of first hospitalization in the VA (1990–1996) or both and found no substantial differences from the full model (RR = 0.70, 95%CI = 0.66–0.74; RR = 0.75, 95% = 0.71–0.80; RR = 0.72, 95% CI = 0.68–0.77, respectively *vs.* RR = 0.74, 95%CI = 0.71–0.78, full model).

## Discussion

Using a case-control study from Lombardy in Italy, and a nested-case control study from a cohort of US Veteran Affairs hospital inpatients, we found a strongly reduced risk of lung cancer in subjects with a personal history of mood disorders. Participants with a family history of mood disorders also had a similar inverse association with lung cancer risk, even in the absence of personal mood disorders. The inverse association was stronger for subjects who had both personal and family history of mood disorders.

Previous studies on the relationship between lung cancer and mood disorders have been mixed. Most prospective investigations examining this relationship, particularly major depression diagnosis, have not identified an association with lung cancer risk [9], [10], [11], [12], [13], [14], [15], [16], while one study [17], with 240 lung cancer cases, found a positive association. These studies may have been affected by the small sample size (only 3 studies [12], [13], [17] included more than 65 cases with prior mood disorder diagnosis). Moreover, most studies did not take into account potential confounders, such as tobacco smoking or COPD [12], [13], [15], [17], allowed for concurrent diagnoses of mood disorders and lung cancer [13] or used different ICD codes corresponding to broader and possibly milder forms of mental disorders [12], [17].

The relationship with both personal and family history of mood disorders and lung cancer suggests that genetic, epigenetic factors or shared environment could be plausible explanations. Indeed, mood disorders have been associated with genetic effects [23], environmental factors [4] or a combination of the two [24].

Treatment for mood disorders may have an effect on lung cancer risk, possibly through the interaction between the use of early generation antidepressants and the inhibition of pro-inflammatory pathways [25] or cytochrome p450 enzymes known to activate carcinogens in tobacco [26]. However, some studies [27], [28] reported that early antidepressant use is associated with increased cancer risk, suggesting that the interplay between smoking and medication, if any, is not straightforward. Also, serotonin appears to stimulate the growth of certain lung cancers [29], [30], [31], [32], [33]. Lowered serotonin levels in mood disorders have been reported both in the central nervous system [34] and in the periphery [35] with possible implications in lung cancer risk.

Mild depression may make individuals less prone to pursue medical assistance [36], with resulting underestimation of mood disorders. However, this should affect all subjects, regardless of future lung cancer diagnosis. Resistance to seek medical care in depressed people may also delay lung cancer diagnosis, but given the inevitable progression and eventual hospitalization, recording of this aggressive disease is a virtual certainty. It can also be argued that subjects with significant mood disorders may seek medical attention on a more frequent basis. Surveillance bias, where lung cancer diagnosis is identified more often in individuals previously followed up due to mood disorder diagnosis would result in a positive association and not inverse, as our study reports, although more surveillance could also result in more frequent smoking cessation counseling that might lessen future cancer rates. Moreover, access to healthcare should not constitute a barrier to identification of a diagnosis of mood disorders in either Italy (which enjoys universal health care) or the US VA System (generally free access for Veterans). While the VA study was based on inpatient data potentially favoring more severe forms of mood disorders, the EAGLE study should have also captured moderate diagnoses treated on an outpatient basis. However, in Italy there is a low propensity for individuals to reveal details of their personal and emotional lives and only a small percentage of those suffering from emotional or mental health problems consult a medical professional [37]. Thus, the subjects with self-reported mood disorders in EAGLE may reflect those with more severe diseases similar to those requiring hospitalization as in the VA study.

A potential issue is that some emotional and cognitive signs of mood disorders (e.g. weight loss, sleep perturbation and fatigue) could derive from pre-clinical manifestations of lung cancer itself [2]. We addressed this issue by excluding subjects with a discharge record for any disease (in the VA study) or mood disorders (in EAGLE) within a year from the cancer diagnosis.

Another concern is that people with mood disorders would experience increased mortality due to comorbid conditions such as cardiovascular disease or suicide [4], [38], and this would be reflected in an inverse association with cancer. However, further adjustment for stroke and ischemic heart disease did not modify the results, suggesting competing mortality from these sources cannot account for the observation.

Our research had several important strengths: although not fully comparable, both studies represented large populations with standardized access to medical care and different epidemiological designs. The VA cohort study featured extended follow-up among males and data on multiple medical conditions while the EAGLE case-control study considered both personal and family history of mood disorders, as well as psychometric scores for mood disorder symptoms. In addition, while one study design was based on self-reported questionnaire data, the other was based on medical records; both resulted in similar findings with high statistical significance. However, the results may only be generalizable to men, as women were not included in the VA cohort study analysis and were less commonly represented in the EAGLE case-control study.

Although we present the largest effort to date to evaluate the association between a previous history of mood disorders and risk of incident lung cancer, our work has several limitations. Misclassification or under-reporting of personal or family history of mood disorders, particularly in EAGLE, where severe depression requiring medication or hospitalization was the inclusion criterion, cannot be completely excluded. However, any such misclassification or under-reporting would probably be nondifferential.

The self-reported mood disorders in EAGLE may be subject to recall bias. However, the self-reported history of mood disorders among controls (91.7% of whom recalled their date of diagnosis or inpatient mood disorders care) was strongly (P<0.0001, Wald test) positively correlated with the CES-D and HADS scores, suggesting that a self-reported history of mood disorders does reflect a past mood disorder diagnosis. Moreover, the prevalence of mood disorders among EAGLE controls in the Lombardy region (9.1% overall, and 7.0% and 15.9% among males and females, respectively) is very similar to the lifetime prevalence of any mood disorders in Italy's non-institutionalized adult population during 1998 (11.2% overall, and 7.2% and 14.9% among males and females, respectively) [37]. Finally, the VA study was based on discharge records, with no risk of recall bias.

Smoking could be an important confounder and/or effect modifier of mood disorders-lung cancer risk associations [39]. Our results show a suggestive, but not significant, interaction between a personal history of mood disorders and smoking status in EAGLE. In fact, the negative association between mood disorders and lung cancer risk was evident in current and former smokers, but not in never smokers, although this last category included only a small number of cases. Similarly, in the analyses of other cancers in the VA study, we found that mood disorders-related protection was more frequent in smoking-related cancers than in those less strongly associated with tobacco smoking (Table S6). However, in the VA study, subjects without smoking-related conditions showed a stronger risk reduction, although we cannot exclude that some smokers were included in this group. Moreover, in the VA study, medical conditions used as surrogate variables for smoking habits or alcohol consumption likely underestimate the actual presence of these exposures. Nonetheless, if these factors were decisive confounders then statistical adjustment for these surrogate variables should decrease the resultant effect estimates, but no major changes were observed. In the EAGLE study we were able to use individual smoking data to directly take into account smoking, and the strength of the inverse association was increased upon adjustment for detailed smoking and alcohol data. Finally, we cannot exclude that cigarette smoking could be used as “self-medication” for mood disorders and in this case, the “non-mood disorders” group used as reference for the association might include some milder forms of mood disorders “treated” by smoking. Since smoking is a strong risk factor for lung cancer and residual confounding from smoking can never be ruled out, follow-up in subjects with other smoking related conditions and in larger samples of non-smoking lung cancer patients is warranted.

In conclusion, using data from two different populations and study designs, we found an inverse association between lung cancer risk and personal or family history of mood disorders. This replicated finding could suggest a new insight in the development of these two widespread and debilitating diseases, although the association could have been affected by tobacco smoking. Further large-scale laboratory and human population and behavior research is necessary to clarify the complex interplay among smoking behavior, inherited susceptibility, mood disorders and cancer risk.

## Materials and Methods

### Ethics Statement

The Environment And Genetics in Lung cancer Etiology (EAGLE) study was approved by the Institutional Review Board (IRB) of each participating hospital and The University of Milan in Italy and by the National Cancer Institute, NIH, in Bethesda, MD. All subjects provided written consent. A detailed description and link to the respective hospitals is available on the EAGLE website (http://dceg.cancer.gov/eagle). Since no personal identifiers were associated with the Veterans Affairs study existing database, and we had no contact with the subjects, the National Institutes of Health (NIH) Office of Human Subjects Research granted us exemption from the Institutional Review and an informed consent waiver.

### Study populations

#### EAGLE study (http://eagle.cancer.gov)

The EAGLE study design and related investigations have been previously described [18]. Briefly, EAGLE enrolled 2,100 incident primary lung cancer cases and 2,120 population-based healthy controls, 35–79 years old, in Italy's Lombardy region, between April 2002 and June 2005. Lung cancer diagnoses were confirmed histopathologically in 95% of cases and by imaging and clinical charts in the remaining 5%. Controls were randomly selected from the Lombardy Regional Health Service population database and frequency matched to cases by age (5-year classes), sex and area of residence. The response rate was 86.6% and 72.4% for eligible cases and controls, respectively.

#### VA study

Patients from the VA Department were selected from computerized discharge records for inpatient visits from the Patient Treatment File from July 1, 1969 to September 30, 1996 at 142 US VA hospitals. These subjects derived from approximately 30 million US veterans eligible for admission to VA hospitals during the study period [40]. To reduce the risk of reverse causality, follow-up began one year after the date of the first hospital discharge for any condition and continued until the end of the observation period, diagnosis of any cancer, or death, whichever occurred first. Dates of death were identified by record linkage to the Social Security Administration Death Master Files [41] by the US Department of VA, prior to granting the investigators access to the data. Our study included 3,669,244 white males, age 18 or older without a prior diagnosis of malignancy if they were hospitalized at least once during the study period, were cancer free during the first year of follow-up and survived at least 1 year after the initial visit. Women and non-whites (due to small numbers), non-veterans and those with documented cancer or death during the first year of follow-up were excluded.

### Exposure ascertainment

#### EAGLE study

In EAGLE, we ascertained a personal history of mood disorders by asking: “Have you ever been told by a doctor that you had severe depression requiring medication or hospitalization?” and “How old were you or in what year was this condition first diagnosed?”. We cannot rule out that EAGLE's participants with depression had or developed a broader mood disorder diagnosis. For example, a diagnostic change from depression to bipolar illness of about 1% per year is expected [42]. Thus, for consistency, we defined depression as “mood disorders” throughout the paper.

A family history of mood disorders was ascertained from the study subjects for each first-degree relative (mother, father, siblings, and children) with the same two questions. The number of siblings in the families ranged from 0 to 18, with a mode of 3 and with 10% with 7 or more siblings. As there were only 16 cases and 17 controls reporting more than one sibling with mood disorders, we defined the family history in siblings, “yes” as having any affected sibling in the family. Similarly, the number of children in the families ranged from 0 to 10, with a mode of 2 and with 7% with 4 or more children. We defined the family history in children as we defined family history in siblings. Families who had any relative with mood disorder diagnosis were defined as “yes”. Subjects with missing information for these questions were assigned to “no”. In a sensitivity analysis we excluded all cases (28.7%) and controls (26.1%) with missing information on family history of mood disorders and observed very similar results. Reported results are based on the entire sample.

The questionnaire provided demographic characteristics (i.e., sex, educational level, marital status), detailed personal smoking history (e.g., number of cigarettes/day, age at initiation, duration, passive smoking and quitting history), and smoking habits of first-degree relatives. Smoking status was categorized as never (smoked less than 100 cigarettes during lifetime), former (quit smoking at least six months or more before interview), and current smokers (still smoking or quit less than six months before interview). We computed the average consumption of alcohol in grams/day and obtained a score for the FTND [22]. Personal symptoms of depression and anxiety more than a year prior to enrollment were evaluated through psychometric measures, i.e., the CES-D [20] and the HADS [21].

We excluded 179 (4.3%) EAGLE participants who did not respond to questions related to personal history of mood disorders, and one case with a date of mood disorder diagnosis less than one year before enrollment in the study. The proportion of excluded cases (n = 161, 7.6%) and controls (n = 18, 0.8%) mirrored non-response rates in the whole questionnaire (7.4% and 0.2%, for cases and controls, respectively). The distribution of the major risk factors for lung cancer (i.e., smoking status, cigarette pack-years, alcohol consumption, age, sex, educational level, and marital status) did not significantly differ between nonresponders and responders to the depression/anxiety psychometric scores. There was no evidence of heterogeneity by case status based on the ability to recall the diagnosis' date of mood disorders (*P* = 0.83, Wald test).

#### VA study

In the VA study, we assessed cancer incidence, personal history of mood disorders and related medical conditions based on the ICD8-Adapted (ICD8-A, from 1969 to 1979) and ICD9-Clinical Modification (ICD9-CM, from 1980 to 1996) [43] revisions. The description of the conditions is reported in Table S8.

### Statistical analyses

#### EAGLE study

Odds ratios (ORs) and 95% confidence intervals (CIs) were estimated using unconditional logistic regression adjusted for age, sex, residence, weighted average grams per day of alcohol consumption, educational level, marital status and smoking. Smoking adjustment included smoking status (categorized as never [smoking <100 cigarettes in a lifetime], former [quit smoking ≥6 months before interview], or current), smoking duration, cigarettes per day, years since quitting (in former smokers), and exposure to environmental tobacco smoke (during childhood, adulthood and at work, in never smokers only). Further adjustment for family history of smoking, body mass index, and history of asthma did not alter estimates, so they were not included in the final model. Interactions between covariates in the adjusted model, history of mood disorders and lung cancer risk were evaluated using the LRT. Stratified analyses were performed by smoking status (current, former, and never smokers) and sex. Homogeneity among histologic and grade specific lung cancer risks was evaluated using the Wald test. We used SAS software, version 9.1 (SAS Institute Inc., Cary, North Carolina).

#### VA study

Relative risks (RR) and 95% CIs for lung cancer incidence in the VA study sample were calculated with Poisson regression [44], using Epicure AMFIT 2.0 (HiroSoft International Corp, Seattle, Washington). Person-years were stratified by categories of attained age (<40, 40–49, 50–59, 60–69, 70–79, *≥*80 years), calendar-year (1969–1974, 1975–1979, 1980–1984, 1985–1989, 1990–1996), hospital visits during the follow up period (1–2, 3–4, ≥5 visits), time between study entry and exit (2–3, 4–5, 6–9, 10–14, ≥15 years), occurrence (yes/no) of chronic obstructive pulmonary disease (COPD), drug dependence and abuse, alcohol-related diagnoses, and schizophrenia. All variables, except number of hospital visits, were treated as time-dependent. To account for potential changes in variable definitions from ICD8-A (1969–1979) to ICD9-CM (1980–1996) periods, we stratified the results by these two calendar periods. Hospital admission date was used as the cancer diagnosis date, and hospital discharge date was used for all other diagnoses.

No direct measurements of smoking or alcohol consumption were available. As surrogates, we used ICD8-A and ICD9-CM diagnostic codes for COPD and drug dependence and abuse, as well as alcohol related diagnoses, respectively. Further adjustment for schizophrenic disorders, often associated with mood disorders, and for ischemic heart disease and stroke was also performed. Table S8 presents the ICD8-A and ICD9-CM discharge codes used to define the relevant covariates.

## Supporting Information

Table S1 **Unadjusted odds ratios (95% confidence intervals) of lung cancer by the variables used in the multivariate analyses in the EAGLE Study, Italy, 2002–2005.**
(DOC)

Table S2 **Numbers of cases and controls and risk estimates for lung cancer by smoking status and categories of mood disorders, EAGLE Study, Italy, 2002–2005.**
(DOC)

Table S3 **Numbers and percentages of cases and controls with and without personal history of mood disorders and risk estimates for lung cancer by categories of cigarette pack years, EAGLE Study, Italy, 2002–2005.**
(DOC)

Table S4 **Numbers and percentages of cases and controls and risk estimates for lung cancer by gender and categories of mood disorders, EAGLE Study, Italy, 2002–2005.**
(DOC)

Table S5 **Numbers and percentages of lung cancer cases (n = 1,939) with and without personal or family history of mood disorders and Wald tests for homogeneity by categories of histology and tumor grade, EAGLE Study, Italy, 2002–2005.**
(DOC)

Table S6 **Relative risks and 95% confidence intervals for cancer incidence with and without history of mood disorders in the United States Veterans Affairs Inpatient Cohort: White males with at least one hospital admission between July 1, 1969, and September 30, 1996.**
(DOC)

Table S7 **Unadjusted relative risks (95% confidence intervals) for lung cancer by the variables used in the multivariate analyses in the United States Veterans Affairs Inpatient Cohort Study: White males (n = 3,669,224) with at least one hospital admission, and were followed for more than one year, between July 1, 1969 and September 30, 1996.**
(DOC)

Table S8 **International Classification of Disease codes for exposures and potential confounders, United States Veterans Affairs Inpatient Cohort: White males (n = 3,669,224) with at least one hospital admission between July 1, 1969 and September 30, 1996.**
(DOC)
