# Supplementary material for: Mood Disorders and Risk of Lung Cancer in the EAGLE Case-Control Study and in the U.S. Veterans Affairs Inpatient Cohort
Source: PLoS One. 2012 Aug 7;7(8):e42945. doi: 10.1371/journal.pone.0042945 (PMC3413657; doi:10.1371/journal.pone.0042945)
Supplement: Table S1 — Unadjusted odds ratios (95% confidence intervals) of lung cancer by the variables used in the multivariate analyses in the EAGLE Study, Italy, 2002–2005. (DOC) [file pone.0042945.s001.doc]

**TABLE S1.** Unadjusted odds ratios (95% confidence intervals) of lung cancer by the variables used in the multivariate analyses in the EAGLE Study, Italy, 2002-2005.

| **Characteristics** |  | **Lung cancer cases** (n=1,939) |  | **Controls** (n=2,102) |  | **Unadjusted model** |
| --- | --- | --- | --- | --- | --- | --- |
|  |  | n (%) |  | n (%) |  | OR (95% CI) |
| **Gender** |  |  |  |  |  |  |
| Males |  | 1,532 (79.0) |  | 1,606 (76.4) |  | 1.16 (1.00-1.35) |
| Females |  | 407 (21.0) |  | 496 (23.6) |  | 1.00 |
| **Age (years)** |  |  |  |  |  |  |
| 30-39 |  | 12 (0.6) |  | 17 (0.8) |  | 1.06 (0.48-2.36) |
| 40-49 |  | 66 (3.4) |  | 99 (4.7) |  | 1.00 |
| 50-59 |  | 344 (17.7) |  | 424 (20.2) |  | 1.22 (0.86-1.71) |
| 60-69 |  | 765 (39.5) |  | 851 (40.5) |  | 1.35 (0.97-1.87) |
| 70-80 |  | 752 (38.8) |  | 711 (33.8) |  | 1.59 (1.14-2.20) |
| **Residence** |  |  |  |  |  |  |
| Brescia |  | 247 (12.7) |  | 247 (11.8) |  | 1.18 (0.89-1.55) |
| Milano |  | 1,275 (65.8) |  | 1,425 (67.8) |  | 1.05 (0.84-1.32) |
| Monza |  | 132 (6.8) |  | 117 (5.6) |  | 1.33 (0.96-1.84) |
| Pavia |  | 128 (6.6) |  | 128 (6.1) |  | 1.18 (0.85-1.63) |
| Varese |  | 157 (8.1) |  | 185 (8.8) |  | 1.00 |
| **Personal history of mood disorders** |  |  |  |  |  |  |
| Yes |  | 121 (6.2) |  | 192 (9.1) |  | 0.66 (0.52-0.84) |
| No |  | 1,818 (93.8) |  | 1,910 (90.9) |  | 1.00 |
| **Any family history of mood disorders** |  |  |  |  |  |  |
| Yes |  | 223 (11.5) |  | 345 (16.4) |  | 0.66 (0.55-0.79) |
| No/Unknown |  | 1,716 (88.5) |  | 1,757 (38.6) |  | 1.00 |
| **Cigarette status (lifetime)** |  |  |  |  |  |  |
| Never |  | 132 (6.8) |  | 679 (32.3) |  | 1.00 |
| Former |  | 838 (43.2) |  | 902 (42.9) |  | 4.78 (3.88-5.89) |
| Current |  | 969 (50.0) |  | 521 (24.8) |  | 9.57 (7.72-11.86) |
| **Cigarette intensity (packs/day) ^a^** |  | 1.00 (0.75-1.50) |  | 0.75 (0.48-1.00) |  | 3.39 (2.90-3.97) |
| **Cigarette duration (years) ^a^** |  | 44.0 (36.0-52.0) |  | 33.0 (21.0-44.0) |  | 1.06 (1.05-1.07) |
| **Years since quitting cigarettes ^a^** |  | 10.0 (3.0-19.0) |  | 20.0 (11.0-30.0) |  | 0.95 (0.94-0.95) |
| **Alcohol (grams)** |  |  |  |  |  |  |
| 0-4.9 g/day |  | 358 (20.5) |  | 475 (23.2) |  | 0.78 (0.65-0.95) |
| 5-14.9 g/day |  | 252 (14.4) |  | 405 (19.8) |  | 1.00 |
| 15-29.9 g/day |  | 436 (24.9) |  | 506 (24.7) |  | 0.90 (0.75-1.08) |
| 30-59.9 g/day |  | 523 (29.9) |  | 572 (27.9) |  | 0.95 (0.80-1.14) |
| >=60 g/day |  | 181 (10.3) |  | 90 (4.4) |  | 2.09 (1.58-2.78) |
| **Education level** |  |  |  |  |  |  |
| Non-educated |  | 112 (5.8) |  | 89 (4.2) |  | 3.24 (2.26-4.65) |
| Elementary school |  | 752 (38.8) |  | 572 (27.2) |  | 3.38 (2.63-4.36) |
| Middle/High School |  | 974 (50.3) |  | 1,181 (56.2) |  | 2.12 (1.66-2.71) |
| University Degree |  | 100 (5.2) |  | 260 (12.4) |  | 1.00 |
| **Marital status** |  |  |  |  |  |  |
| Married or Cohabitating |  | 1,493 (77.0) |  | 1,737 (82.6) |  | 1.00 |
| Single/ Separated/Widow/ Divorced |  | 446 (23.0) |  | 365 (17.4) |  | 1.42 (1.22-1.66) |

**Abbreviation:** EAGLE, Environment And Genetics in Lung cancer Etiology.

^a^ Median (inter-quartile range).

**^b^** “Non-educated” subjects are those who did not complete the elementary school.

**Note:** Numbers of participants may not sum to total due to missing data.
