# Supplementary material for: Mood Disorders and Risk of Lung Cancer in the EAGLE Case-Control Study and in the U.S. Veterans Affairs Inpatient Cohort
Source: PLoS One. 2012 Aug 7;7(8):e42945. doi: 10.1371/journal.pone.0042945 (PMC3413657; doi:10.1371/journal.pone.0042945)
Supplement: Table S2 — Numbers of cases and controls and risk estimates for lung cancer by smoking status and categories of mood disorders, EAGLE Study, Italy, 2002–2005. (DOC) [file pone.0042945.s002.doc]

**TABLE S2.** Numbers of cases and controls and risk estimates for lung cancer by smoking status and categories of mood disorders, EAGLE Study, Italy, 2002-2005.

| **Mood** |  |  | | |  |  | | | | |  |  | | | |  |  | | | | | |  | |  | |
| --- | --- | --- | --- | --- | --- | --- | --- | --- | --- | --- | --- | --- | --- | --- | --- | --- | --- | --- | --- | --- | --- | --- | --- | --- | --- | --- |
| **disorders status** |  | **Overall ^a^** | | |  | **Current smokers ^b^** | | | | |  | **Former smokers ^b^** | | | |  | **Never smokers ^c^** | | | | | |  | | **P Interaction ^d^** | |
|  |  | Case | Control | OR |  | Case | Control | | | OR |  | Case | Control | | OR |  | Case | | Control | | | OR |  | |  | |
|  |  | (n=1746) | (n=2046) | (95% CI) |  | (n=857) | (n=497) | | | (95% CI) |  | (n=774) | (n=886) | | (95% CI) |  | (n=101) | | (n=595) | | | (95% CI) |  | |  | |
| Personal history |  | 109 | 189 | 0.59 |  | 58 | 46 | | | 0.56 |  | 36 | 69 | | 0.48 |  | 14 | | 67 | | | 0.97 |  | | 0.26 | |
|  |  |  |  | (0.44-0.79) |  |  |  | | | (0.36-0.88) |  |  |  | | (0.29-0.79) |  |  | |  | | | (0.50-1.88) |  | |  | |
| Family history |  | 201 | 337 | 0.62 |  | 100 | 92 | | | 0.53 |  | 84 | 147 | | 0.68 |  | 15 | | 84 | | | 0.89 |  | | 0.11 | |
|  |  |  |  | (0.50-0.77) |  |  |  | | | (0.38-0.75) |  |  |  | | (0.49-0.95) |  |  | |  | | | (0.47-1.69) |  | |  | |
| Personal with no family history |  | 78 | 124 | 0.65 |  | 44 | 30 | | | 0.64 |  | 25 | 44 | | 0.55 |  | 9 | | 45 | | | 0.97 |  | | 0.61 | |
|  |  |  |  | (0.46-0.92) |  |  |  | | | (0.38-1.08) |  |  |  | | (0.30-1.01) |  |  | |  | | | (0.43-2.17) |  | |  | |
| Family with no personal history |  | 170 | 272 | 0.67 |  | 86 | 76 | | | 0.57 |  | 73 | 122 | | 0.77 |  | 10 | | 62 | | | 0.86 |  | | 0.24 | |
|  |  |  |  | (0.53-0.85) |  |  |  | | | (0.40-0.83) |  |  |  | | (0.54-1.11) |  |  | |  | | | (0.40-1.84) |  | |  | |
| Both personal & family history |  | 31 | 65 | 0.51 |  | 14 | 16 | | | 0.45 |  | 11 | 25 | | 0.40 |  | 5 | | 22 | | | 0.98 |  | | 0.18 | |
|  |  |  |  | (0.31-0.85) |  |  | |  | (0.20-1.01) | |  |  |  | (0.17-0.91) | |  | |  | |  | (0.35-2.78) | | |  | |  |

**Abbreviations:** OR, odds ratio; CI, confidence interval; EAGLE, Environment And Genetics in Lung cancer Etiology.

^a^ Adjusted for sex, age, residence, time weighted mean alcohol consumption (grams/day), education level, marital status and smoking status.

^b^ Additionally adjusted for years smoked and mean cigarettes smoked per day (current and former smokers), years since quitting (former smokers).

^c^ Additionally adjusted for exposure to environmental tobacco smoke in childhood, adulthood and at work (never-smokers).

^d^ Likelihood ratio test P-value for smoking status (current, former and never) interaction.

**Note:** Numbers of participants may not sum to total due to missing data.
