# Supplementary material for: Mood Disorders and Risk of Lung Cancer in the EAGLE Case-Control Study and in the U.S. Veterans Affairs Inpatient Cohort
Source: PLoS One. 2012 Aug 7;7(8):e42945. doi: 10.1371/journal.pone.0042945 (PMC3413657; doi:10.1371/journal.pone.0042945)
Supplement: Table S3 — Numbers and percentages of cases and controls with and without personal history of mood disorders and risk estimates for lung cancer by categories of cigarette pack years, EAGLE Study, Italy, 2002–2005. (DOC) [file pone.0042945.s003.doc]

**TABLE S3.** Numbers and percentages of cases and controls with and without personal history of mood disorders and risk estimates for lung cancer by categories of cigarette pack years, EAGLE Study, Italy, 2002–2005.

| **Cigarette pack-years**  **(smokers’ quartiles) ^a^** |  | **Personal history of mood disorders** | | | | |  |  |  |  |
| --- | --- | --- | --- | --- | --- | --- | --- | --- | --- | --- |
|  |  | **Lung cancer cases** | |  | **Controls** | |  | **Minimally Adjusted ^b^** |  | **Fully Adjusted ^c^** |
|  |  | Yes | No |  | Yes | No |  |  |  |  |
|  |  | (n=121) | (n=1,815) |  | (n=192) | (n=1,908) |  |  |  |  |
|  |  | n (%) | n (%) |  | n (%) | n (%) |  | OR (95% CI) |  | OR (95% CI) |
| Never-smokers |  | 17 (14.0) | 115 (6.3) |  | 75 (39.1) | 604 (31.7) |  | 0.89 (0.49-1.61) |  | 0.97 (0.50-1.88) |
| 0.1 - 19.9 |  | 14 (11.6) | 190 (10.5) |  | 46 (24.0) | 557 (39.2) |  | 0.76 (0.40-1.45) |  | 0.76 (0.39-1.48) |
| 20.0 - 35.9 |  | 18 (14.9 ) | 378 (20.8 ) |  | 30 (15.6 ) | 339 (17.8 ) |  | 0.47 (0.25 -0.88) |  | 0.42 (0.22 -0.82 ) |
| 36.0 - 52.9 |  | 30 (24.8 ) | 534 (29.4 ) |  | 23 (12.0 ) | 261 (13.7 ) |  | 0.53 (0.29 -0.95 ) |  | 0.51 (0.27 -0.94 ) |
| >=53.0 |  | 42 (34.7) | 598 (32.9) |  | 18 (9.4) | 147 (7.7) |  | 0.53 (0.29-0.95) |  | 0.47 (0.25-0.89) |

**Abbreviations:** OR, odds ratio; CI, confidence interval; EAGLE, Environment And Genetics in Lung cancer Etiology.

^a^ Quartiles categories for smokers’ pack-years.

^b^ Adjusted for sex, age and residence.

^c^ Adjusted for sex, age, residence, time weighted mean alcohol consumption (grams/day), education level and marital status. ORs are additionally adjusted for years since quitting cigarettes (in smokers) or exposure to environmental tobacco smoke in childhood, adulthood and at work (in never-smokers).

**Note:** Numbers of participants may not sum to total due to missing data.
