# Supplementary material for: Mood Disorders and Risk of Lung Cancer in the EAGLE Case-Control Study and in the U.S. Veterans Affairs Inpatient Cohort
Source: PLoS One. 2012 Aug 7;7(8):e42945. doi: 10.1371/journal.pone.0042945 (PMC3413657; doi:10.1371/journal.pone.0042945)
Supplement: Table S4 — Numbers and percentages of cases and controls and risk estimates for lung cancer by gender and categories of mood disorders, EAGLE Study, Italy, 2002–2005. (DOC) [file pone.0042945.s004.doc]

**TABLE S4.** Numbers and percentages of cases and controls and risk estimates for lung cancer by gender and categories of mood disorders, EAGLE Study, Italy, 2002–2005.

|  |  | **History of mood disorders** | | | | |  |  |
| --- | --- | --- | --- | --- | --- | --- | --- | --- |
|  |  | **Lung cancer cases** | |  | **Controls** | |  |  |
| **Mood disorders status by gender** |  | **(n=1,746)** | |  | **(n=2,046)** | |  | **Adjusted model ^a^** |
|  |  | Yes | No |  | Yes | No |  |  |
|  |  | n (%) | n (%) |  | n (%) | n (%) |  | OR (95% CI) |
| Males |  |  |  |  |  |  |  |  |
| Personal history |  | 71 (5.1) | 1,317 (94.9) |  | 111 (7.1) | 1,456 (92.9) |  | 0.61 (0.42-0.89) |
| Family history |  | 148 (10.7) | 1,240 (89.3) |  | 241 (15.4) | 1,326 (84.6) |  | 0.61 (0.47-0.80) |
| Both personal & family history |  | 19 (1.4) | 1,369 (98.6) |  | 37 (2.4) | 1,530 (97.6) |  | 0.44 (0.23-0.85) |
| Females |  |  |  |  |  |  |  |  |
| Personal history |  | 38 (10.6) | 320 (89.4) |  | 78 (16.3) | 401 (83.7) |  | 0.58 (0.36-0.93) |
| Family history |  | 53 (14.8) | 305 (85.2) |  | 96 (20.0) | 383 (80.0) |  | 0.66 (0.43-1.01) |
| Both personal & family history |  | 12 (3.4) | 346 (96.7) |  | 28 (5.8) | 451 (94.2) |  | 0.67 (0.31-1.45) |

**Abbreviations:** OR, odds ratio; CI, confidence interval; EAGLE, Environment And Genetics in Lung cancer Etiology.

^a^ Adjusted for age, residence, time weighted mean alcohol consumption (grams/day), education level, marital status, smoking status, years smoked, years since quitting and average cigarettes smoked per day.

**Note:** Numbers of participants may not sum to total due to missing data.
