# Supplementary material for: Mood Disorders and Risk of Lung Cancer in the EAGLE Case-Control Study and in the U.S. Veterans Affairs Inpatient Cohort
Source: PLoS One. 2012 Aug 7;7(8):e42945. doi: 10.1371/journal.pone.0042945 (PMC3413657; doi:10.1371/journal.pone.0042945)
Supplement: Table S5 — Numbers and percentages of lung cancer cases (n = 1,939) with and without personal or family history of mood disorders and Wald tests for homogeneity by categories of histology and tumor grade, EAGLE Study, Italy, 2002–2005. (DOC) [file pone.0042945.s005.doc]

**TABLE S5.** Numbers and percentages of lung cancer cases (n = 1,939) with and without personal or family history of mood disorders and Wald tests for homogeneity by categories of histology and tumor grade, EAGLE Study, Italy, 2002–2005.

| **Tumor characteristics** |  | **Personal mood disorders** | |  | **Family mood disorders** | |
| --- | --- | --- | --- | --- | --- | --- |
|  |  | Yes | No |  | Yes | No / Unknown |
|  |  | (n=121) | (n=1,818) |  | (n=223) | (n=1,716) |
|  |  | n (%) | n (%) |  | n (%) | n (%) |
| **Type of Lung Tumor** |  | P = 0.52 ^a^ | |  | P = 0.17 ^a^ | |
| Adenocarcinoma |  | 54 (44.6) | 685 (37.7) |  | 83 (37.2) | 656 (38.2) |
| Squamous |  | 32 (26.4) | 472 (26.0) |  | 50 (22.4) | 454 (26.5) |
| Small Cell |  | 10 (8.3) | 185 (10.2) |  | 29 (13.0) | 166 (9.7) |
| Large Cell |  | 5 (4.1) | 84 (4.6) |  | 15 (6.7) | 74 (4.3) |
| Other Lung Histologies |  | 16 (13.2) | 293 (16.1) |  | 38 (17.0) | 271 (15.8) |
| Missing Histology Information |  | 4 (3.3) | 99 (5.4) |  | 8 (3.6) | 95 (5.5) |
| **Tumor Grade** |  | P = 0.52 ^b^ | |  | P = 0.49 ^b^ | |
| Well Differentiated |  | 4 (3.3) | 62 (3.4) |  | 13 (5.8) | 53 (3.1) |
| Moderately Differentiated /Intermediate |  | 22 (18.2) | 302 (16.6) |  | 31 (13.9) | 293 (17.1) |
| Undifferentiated /Anaplastic |  | 50 (41.3) | 683 (37.6) |  | 88 (39.5) | 645 (37.6) |
| Missing Grade Information |  | 45 (37.2) | 771 (42.4) |  | 91 (40.8) | 725 (42.2) |

^a^ Wald test for homogeneity, four degrees of freedom.

^b^ Wald test for homogeneity, two degrees of freedom.

**Note:** Numbers of participants may not sum to total due to missing data.
