# Supplementary material for: Mood Disorders and Risk of Lung Cancer in the EAGLE Case-Control Study and in the U.S. Veterans Affairs Inpatient Cohort
Source: PLoS One. 2012 Aug 7;7(8):e42945. doi: 10.1371/journal.pone.0042945 (PMC3413657; doi:10.1371/journal.pone.0042945)
Supplement: Table S6 — Relative risks and 95% confidence intervals for cancer incidence with and without history of mood disorders in the United States Veterans Affairs Inpatient Cohort: White males with at least one hospital admission between July 1, 1969, and September 30, 1996. (DOC) [file pone.0042945.s006.doc]

**TABLE S6.** Relative risks and 95% confidence intervals for cancer incidence with and without history of mood disorders in the United States Veterans Affairs Inpatient Cohort: White males with at least one hospital admission between July 1, 1969, and September 30, 1996.

|  |  | **History of Mood Disorders ^a^** | | | | |  |  |
| --- | --- | --- | --- | --- | --- | --- | --- | --- |
|  |  | **With each specific cancer diagnosis** | |  | **Without each specific cancer diagnosis** | |  |  |
| **Cancer type** |  | **(number)** | |  | **(person-years)** | |  | **Adjusted model ^b^** |
|  |  | Yes | No |  | Yes | No |  |  |
|  |  | n (%) | n (%) |  |  |  |  | RR (95% CI) |
| Lung ^c^ |  | 2,304 (2.8) | 80,641 (97.2) |  | 177,267 | 3,409,032 |  | 0.74 (0.71 – 0.78) |
| Larynx ^d^ |  | 262 (2.4) | 10,851 (97.6) |  | 179,309 | 3,478,822 |  | 0.61 (0.54 – 0.70) |
| Esophageal ^e^ |  | 133 (2.1) | 6,204 (97.9) |  | 179,438 | 3,483,469 |  | 0.57 (0.47 – 0.68) |
| Bladder ^f^ |  | 493 (2.8) | 16,930 (97.2) |  | 179,078 | 3,472,743 |  | 0.86 (0.78 – 0.94) |
| Colon ^g^ |  | 480 (2.6) | 17,739 (97.4) |  | 179,091 | 3,471,934 |  | 0.83 (0.76 – 0.92) |
| Non Hodgkin Lymphoma ^h^ |  | 312 (3.9) | 7,687 (96.1) |  | 179,259 | 3,481,986 |  | 0.84 (0.74 – 0.96) |
| Prostate ^i^ |  | 1,610 (3.3) | 47,726 (96.7) |  | 177,961 | 3,441,947 |  | 1.00 (0.85 – 1.06) |
| Melanoma ^j^ |  | 165 (4.1) | 3866 (95.9) |  | 179,406 | 3,485,807 |  | 1.13 (0.95 – 1.34) |

**Abbreviations:** RR, relative risk; CI, confidence interval; ICD, International Classification of Disease.

^a^ ICD-8 & ICD-9, code 296; which includes depression and bipolar disease.

^b^ Adjusted for number of visits, age, latency, calendar time, alcohol and drug dependence and abuse, COPD and schizophrenia.

^c^ ICD-8 & ICD-9 code 162

^d^ ICD-8 & ICD-9 code 161

^e^ ICD-8 & ICD-9 code 150

^f^ ICD-8 & ICD-9 code 188

^g^ ICD-8 & ICD-9 code 153

^h^ ICD-8 & ICD-9 code 200

^i^ ICD-8 & ICD-9 code 185

^j^ ICD-8 & ICD-9 code 172

**Note:** Numbers of participants may not sum to total due to missing data.
