# Supplementary material for: Mood Disorders and Risk of Lung Cancer in the EAGLE Case-Control Study and in the U.S. Veterans Affairs Inpatient Cohort
Source: PLoS One. 2012 Aug 7;7(8):e42945. doi: 10.1371/journal.pone.0042945 (PMC3413657; doi:10.1371/journal.pone.0042945)
Supplement: Table S7 — Unadjusted relative risks (95% confidence intervals) for lung cancer by the variables used in the multivariate analyses in the United States Veterans Affairs Inpatient Cohort Study: White males (n = 3,669,224) with at least one hospital admission, and were followed for more than one year, between July 1, 1969 and September 30, 1996. (DOC) [file pone.0042945.s007.doc]

**TABLE S7. Unadjusted relative risks (95% confidence intervals) for lung cancer by the variables used in the multivariate analyses in the United States Veterans Affairs Inpatient Cohort Study: White males (n=3,669,224) with at least one hospital admission, and were followed for more than one year, between July 1, 1969 and September 30, 1996.**

| **Characteristics** |  | | **Lung cancer patients** |  | **Non-cancer patients** |  | **Unadjusted model** |
| --- | --- | --- | --- | --- | --- | --- | --- |
|  |  | | (n=82,945) |  | (n=3,586,299) |  |  |
|  |  | | n (%) |  | Person-years (%) |  | RR (95% CI) |
| **Age at study entry (years)** |  | |  |  |  |  |  |
| < 40 |  | | 2,814 (3.4) |  | 848,653 (23.7) |  | 1.00 |
| 40-49 |  | | 15,031 (18.1) |  | 648,914 (18.1) |  | 8.11 (7.79 - 8.44) |
| 50-59 |  | | 33,545 (40.4) |  | 918,636 (25.6) |  | 14.33 (13.79 - 14.89) |
| 60-69 |  | | 22,801 (27.5) |  | 736,211 (20.5) |  | 17.32 (16.65 - 18.01) |
| 70-79 |  | | 7315 (8.8) |  | 313,907 (8.8) |  | 17.64 (16.89 - 18.42) |
| 80+ |  | | 1439 (1.7) |  | 120,068 (3.3) |  | 11.15 (10.46 - 11.88) |
| **Age at Lung Cancer Diagnosis** |  | |  |  |  |  |  |
| < 40 |  | | 324 (0.4) |  | NA |  | NA |
| 40-49 |  | | 3,197 (3.9) |  | NA |  | NA |
| 50-59 |  | | 19,007 (22.9) |  | NA |  | NA |
| 60-69 |  | | 37,321 (45.0) |  | NA |  | NA |
| 70-79 |  | | 18,788 (22.7) |  | NA |  | NA |
| 80+ |  | | 43,08 (5.2) |  | NA |  | NA |
| **Years of follow-up** |  | |  |  |  |  |  |
| 2–3 |  | | 15,209 (18.3) |  | 398,891 (11.1) |  | 1.00 |
| 4–5 |  | | 12,693 (15.3) |  | 345,377 (9.6) |  | 0.94 (0.92 - 0.96) |
| 6–9 |  | | 24,935 (30.1) |  | 618,959 (17.3) |  | 0.91 (0.89 - 0.93) |
| 10–14 |  | | 16,750 (20.2) |  | 707,444 (19.7) |  | 0.87 (0.85 - 0.89) |
| 15+ |  | | 13,358 (16.1) |  | 1,515,628 (42.3) |  | 0.71 (0.70 - 0.73) |
| **No. of hospital visits** |  | |  |  |  |  |  |
| 1-2 |  | | 17,636 (21.3) |  | 1,797,699 (50.1) |  | 1.00 |
| 3-4 |  | | 25,608 (30.9) |  | 699,150 (19.5) |  | 4.00 (3.92 - 4.07) |
| 5+ |  | | 39,701 (47.9) |  | 1,089,450 (30.4) |  | 3.27 (3.21 - 3.33) |
| **Year of first hospitalization in the VA** | |  |  |  |  |  |  |
| 1969–1974 | |  | 2,724 (3.3) |  | 114,060 (3.2) |  | 1.00 |
| 1975–1979 | |  | 14,247 (17.2) |  | 239,715 (6.7) |  | 1.00 (0.97 - 1.03) |
| 1980–1984 | |  | 19,702 (23.8) |  | 295,373 (8.2) |  | 1.03 (1.00 - 1.06) |
| 1985–1989 | |  | 22,781 (27.5) |  | 330,037 (9.2) |  | 1.03 (1.00 - 1.06) |
| 1990–1996 | |  | 20,491 (24.7) |  | 2,607,114 (72.7) |  | 0.65 (0.63 - 0.67) |
| **Mood disorders ^a^** | |  |  |  |  |  |  |
| Yes | |  | 2,304 (2.8) |  | 177,267 (4.9) |  | 0.75 (0.72 - 0.78) |
| No | |  | 80,827 (97.4) |  | 3,409,032 (95.1) |  | 1.00 |
| **COPD ^b^** | |  |  |  |  |  |  |
| Yes | |  | 26,338 (31.8) |  | 614,518 (17.1) |  | 4.18 (4.12 - 4.24) |
| No | |  | 56,607 (68.2) |  | 2,971,781 (82.9) |  | 1.00 |
| **Alcohol dependence and abuse ^c^** | |  |  |  |  |  |  |
| Yes | |  | 23,903 (28.8) |  | 900,834 (25.1) |  | 1.42 (1.40 - 1.45) |
| No | |  | 59,042 (71.2) |  | 2,685,465 (74.9) |  | 1.00 |
| **Substance dependence and abuse ^d^** | |  |  |  |  |  |  |
| Yes | |  | 1,114 (1.3) |  | 215,291 (6.0) |  | 0.26 (0.25 - 0.28) |
| No | |  | 81,831 (98.7) |  | 3,371,008 (94.0) |  | 1.00 |
| **Schizophrenia ^e^** | |  |  |  |  |  |  |
| Yes | |  | 4,467 (5.4) |  | 216,734 (6.0) |  | 0.70 (0.68 - 0.72) |
| No | |  | 78,478 (94.6) |  | 3,369,565 (94.0) |  | 1.00 |

**Abbreviations:** RR, relative risk; CI, confidence interval; COPD, Chronic Obstructive Pulmonary Disease; ICD, International Classification of Disease; NA, not applicable.

**^a^** ICD-8 & ICD-9, code 296 which includes depression and bipolar I disease.

**^b^** ICD-8 & ICD-9, codes 490-492

**^c^** ICD-8 & ICD-9, codes 291, 303, 305.0, 535.3, 571.0-571.3, 980.0

**^d^** ICD-8 & ICD-9, codes 304-305

**e** ICD-8 & ICD-9, code 295

**Note:** Numbers of participants may not sum to total due to missing data.
