# Supplementary material for: Mood Disorders and Risk of Lung Cancer in the EAGLE Case-Control Study and in the U.S. Veterans Affairs Inpatient Cohort
Source: PLoS One. 2012 Aug 7;7(8):e42945. doi: 10.1371/journal.pone.0042945 (PMC3413657; doi:10.1371/journal.pone.0042945)
Supplement: Table S8 — International Classification of Disease codes for exposures and potential confounders, United States Veterans Affairs Inpatient Cohort: White males (n = 3,669,224) with at least one hospital admission between July 1, 1969 and September 30, 1996. (DOC) [file pone.0042945.s008.doc]

**TABLE S8**. International Classification of Disease codes for exposures and potential confounders, United States Veterans Affairs Inpatient Cohort: White males (n=3,669,224) with at least one hospital admission between July 1, 1969 and September 30, 1996.

| **VA Variable** |  | **Condition** |  | **ICD-8 Codes** |  | **ICD-9 Codes** |
| --- | --- | --- | --- | --- | --- | --- |
| **Exposure** |  |  |  |  |  |  |
| Mood disorders |  | Episodic mood/affective disorders |  | 296 |  | 296 |
| **Potential Confounders** |  |  |  |  |  |  |
| Alcohol |  | Alcohol abuse/dependence, Alcohol-induced mental disorders, Alcoholic gastritis, Chronic liver disease and cirrhosis |  | 291, 303, 571.0, 980.0 |  | 291, 303, 305.0, 535.3, 571.0-571.3, 980.0 |
| Schizophrenia |  | Schizophrenic disorders |  | 295 |  | 295 |
| COPD |  | Chronic airway obstruction, Chronic Bronchitis, Emphysema |  | 490-492 |  | 490-492, 496 |
| Drug dependence and abuse |  | Drug dependence & Nondependent abuse of drugs |  | 304 |  | 304, 305.2-305.9 |

**Abbreviations:** ICD, International Classification of Disease; VA, Veterans Affairs; COPD, Chronic Obstructive Pulmonary Disease.
